# MEttLE: a modelling-based learning environment for undergraduate engineering estimation problem solving

**DOI:** 10.1186/s41039-018-0083-y

**Published:** 2018-11-16

**Authors:** Aditi Kothiyal, Sahana Murthy

**Affiliations:** Interdisciplinary Program in Educational Technology, Indian Institute of Technology Bombay, Mumbai, India

**Keywords:** Estimation, Modelling, Design-based research, Technology-enhanced learning environment

## Abstract

Estimation is an important class of problems that engineering undergraduates must be able to solve. However teaching-learning of estimation is under emphasized in the current engineering curriculum. In this paper, we report on the first cycle of a design-based research project to design a technology-enhanced learning environment (TELE) to help students learn estimation. The TELE includes features such as a progressive higher-order modelling-based structuring of the estimation process, a problem system simulator and metacognitive scaffolds. We performed a lab study and found that learners were able to use the features in the TELE to solve the estimation problem and obtain an order-of-magnitude estimate. Further, learners learned some of the reasoning processes involved in performing estimation and recognized the role of evaluation and the need for practical considerations in estimation. We identified the roles of various features in the TELE for learning these estimation reasoning processes. These results have implications for the redesign of our TELE to improve student learning of estimation.

## Introduction

Engineers routinely make estimates of physical quantities such as power before they begin designing or making (Dym et al. 2005). For example, consider this problem: “You are participating in an electric car race in which you are required to design an electric car of weight 7 kg with wheel diameters of 4 in. that can accelerate at 1 m/s^2^ and traverse a track of 10 m without burning out. Estimate the electrical power needed to achieve this performance and the specifications of the motor you will need”. Engineers often make such estimates and judgements regarding physical quantities, in order to establish the feasibility of a design or narrow down the set of design choices (Dunn-rankin 2001; Shakerin 2006). Estimation allows the designer to begin the design process or move ahead in a situation of low information or resources (Adolphy et al. 2009; Mahajan 2014; Dunn-rankin 2001; Shakerin 2006). Not doing estimation could lead to doing precise calculations which are irrelevant, as a result of which the design would be flawed and unsafe (Raviv et al. 2016). Thus, estimation is very important in engineering.

Synthesizing several definitions of estimation (Linder 1999; Adolphy et al. 2009; Mahajan 2014; Dunn-rankin 2001; Shakerin 2006) for our purposes, we define estimation as *the process of determining approximate values for a physical quantity without the tools of complete information and knowledge*. Making estimates involves the creation of a simplified model (i.e. an equation) for the quantity to be estimated (in the example above, power) (Mahajan 2014) in terms of parameters that it depends on in the given problem situation. This is done by applying engineering principles in real-world situations via practices of engineering such as identifying dominating parameters, quantifying inefficiencies and making approximations and assumptions. Thus, in addition to engineering principles, students need to apply reasoning processes such as comparing and decision-making.

Several practicing engineers, instructors and engineering education researchers have emphasized that students should be trained in estimation (Linder and Flowers 2001; Nachtmann et al. 2003; Shakerin 2006; Adolphy et al. 2009; Raviv et al. 2016). Hence, it is important to improve students’ estimation problem solving. However, recent literature suggests that estimation has been under-emphasized in the current curriculum, as a result of which students are unable to do estimation even after graduating (Raviv et al. 2016). Research comparing the estimation performance of practicing engineers and final year undergraduate students reveals that there is a marked difference between the two groups in the quality of estimates for quantities like drag force and energy (Linder 1999). This suggests that students have little intuition for these quantities. Perhaps, as Ferguson observed (Ferguson 1977), “*The real ‘problem’ of engineering education is the implicit acceptance of the notion that high-status analytic courses are superior to those that encourage the student to develop an intuitive ‘feel’ for the incalculable complexity of engineering practice in the real world.*” Further, there are differences between the characteristics of the learning activities of engineering science and design (Linder and Flowers 2001). The former is primarily well-structured in nature while estimation is ill-structured. It is known that (Jonassen et al. 2006) the ability to solve well-structured problems does not transfer to the ill-structured problems. Thus, the learning that happens in current engineering curricula does not prepare students for estimation. Therefore, it is important to explicitly train students in doing estimation.

A literature survey to identify teaching-learning strategies for estimation showed that while there are some guidelines and activities to teach estimation (Mahajan 2014; Linder and Flowers 2001; Shakerin 2006; Dunn-rankin 2001), there is a dearth of research-based teaching-learning strategies for estimation. Further, estimation has not been formally included in engineering curricula, except for software engineering. This motivates our design-based research (DBR) project to design and evaluate a technology-enhanced learning environment (TELE) for estimation. In this paper, we report on the first cycle of DBR, beginning with the design of our TELE. As estimation requires building a model of the mechanical system in which a quantity needs to be estimated, our solution is a TELE called modelling-based estimation learning environment (MEttLE). In MEttLE, students learn how to estimate a quantity (e.g. power) using a structured estimation process based on model order progression (Swaak et al. 1998; Sun and Looi 2013) and using scaffolds to do estimation. Next, we describe the evaluation of MEttLE with the research goal of exploring what and how learners learned about estimation after interacting with MEttLE. We conducted a lab study wherein learners solved an estimation problem in MEttLE, and we collected multimodal data in order to understand how learners solved the problem, what they learned and how the features of MEttLE supported this learning. Finally, we present our reflections from the first cycle that have implications for identifying the learning mechanisms of estimation and redesign of MEttLE in the second cycle of DBR.

## Literature review

### Estimation

Estimation can be understood as a way of getting insight into engineering problems by “mastering the complexity” (Mahajan 2014) using tools to organize and discard complexity. Estimation has been variously defined as “an analysis to determine all quantities to some level of specificity” (Linder 1999) and “making decisions or selecting from a multitude of options based on incomplete or unavailable details or data” (Shakerin 2006) among others. We believe that each of these definitions is incomplete for our purposes; while the first one does not mention the lack of resources, the second one emphasizes decision-making over determining values. Hence, for the purpose of this paper, we adopt the definition mentioned before, i.e. *the process of determining approximate values for a physical quantity without the tools of complete information and knowledge*.

The purpose of estimation is typically to make a decision that allows one to proceed in the problem solving or design process when faced with lack of information, resources or strategies (Adolphy et al. 2009). In engineering analysis, often an estimate is not only acceptable but more useful than a detailed analysis because it provides useful information about a problem or a design in situations where accurate values are unnecessary, impractical or impossible because of a lack of time, information and/or resources (Linder 1999; Shakerin 2006; Dunn-rankin 2001; Paritosh and Forbus 2003). As Mahajan (2014) puts it, estimation is a process by which we learn to “lower our standards” and create models of the world that we can work with using the conceptual and procedural understanding that we already have. It is the process of solving complex physical problems by relaxing our demands on accuracy, creating a “coarse” model and attempting to understand a situation that we would otherwise have no intuition for.

In order to estimate a quantity, e.g. power, a solver needs to create an equation of power related to the parameters that significantly impact its value in the given problem system (Linder 1999; Mahajan 2014). This is done by creating a model of the real-world system similar to a physical system that is well-understood (Dunn-rankin 2001; Linder 1999). This requires making reasonable assumptions and validating the model by using tests or by relaxing the assumptions. Conceptual knowledge is the foundation for such modelling in addition to techniques such as guessing, divide-and-conquer, abstraction and conservation and using external representations (Linder 1999; Mahajan 2014). Then, the solver needs to create an equation relating power to the parameters that affect it in the model, substitute suitable values for parameters in the equation, calculate and evaluate the estimate. Thus, in addition to conceptual knowledge, solvers need to be able to do modelling by making reasonable assumptions and make judgements regarding numerical values.

### Teaching-learning of estimation and other ill-structured problems

Students’ performance on estimation problems (Linder 1999) shows that they have difficulty in making estimates because they do not have a sound understanding of fundamental engineering concepts. Further, students did not relate the estimates they made to their physical significance, do not have reference values for the quantities they are estimating and have difficulties working with units. In engineering ill-structured problem solving, it has been found that students do not recognize the importance of using different kinds of external representations (visual, verbal and mathematical) and simplifying problem situations (Elger et al. 2003; Adams and Turner 2008). They also face challenges in the epistemic and metacognitive aspects of ill-structured problem solving such as reviewing and revising their solutions, reflecting and changing their methods, and making assumptions and assessing them (Shekoyan 2009). Thus, all these challenges should be supported in a LE for estimation.

Several engineering researchers and practitioners have recognized the importance of helping students explicitly learn how to solve estimation problems (Linder and Flowers 2001; Shakerin 2006; Varma 2009; Malcolm 2013; Mahajan 2017) and offered guidelines for learning activities for rough estimation. Linder recommends teaching the conceptual knowledge of estimation, increasing the number of rough estimation activities done by students and including learning activities that have characteristics similar to those of rough estimation in which students have to select relevant information and balance different types of information. Mahajan uses a five-step approach in his course titled “The Art of Approximation in Science and Engineering” with the steps (a) describe an estimation tool like divide and conquer, (b) illustrate its application with an example from a particular domain, (c) repeat with examples from different domains, (d) provide practice in the usage of a tool in practice problems and (e) present more practice problems without clues as to which tools to use so that students learn to select which tools to apply. Dunn-Rankin suggests that whenever possible, numerical values be tied to everyday physical objects and activities. This helps students develop an intuition for reasonable values for physical quantities or numerical sense which is very important while estimating. Shakerin recommends that students be encouraged to practice estimation and be made aware of its importance through short exercises with everyday objects and activities. While these are all sound guidelines for teaching estimation, these are not based on principles from learning sciences that describe how students learn problem solving and how to support them to overcome the specific challenges in estimation identified above. Thus, more work is needed to derive these teaching guidelines from theory and empirically validate their effectiveness for learning estimation because this will make these guidelines systematic and generalizable and take them beyond heuristics.

The criteria for ill-structured problems have been defined in several places elsewhere, see for example (Shin et al. 2003; Jonassen et al. 2006). According to these criteria, estimation is an ill-structured problem because not all the problem elements are presented, the goals are unclear, there are implicit constraints, there are multiple solutions, multiple solution paths and multiple evaluation criteria, and there is an uncertainty about which concepts, rules or principles to apply. There are many models for solving ill-structured problems available in literature, see for example (Jonassen 2011). To summarize, the steps involved in solving ill-structured problems are defining the problem, gathering relevant information, identifying the sub-goals, developing solutions, assessing alternate solutions and providing arguments for chosen solutions and evaluating the chosen solution. Literature, for example (Jonassen 2011; Ge and Land 2004), has many strategies for supporting the learning of ill-structured problem solving, with scaffolds for each step of problem solving depending on the cognitive requirements of the step. Several researchers have empirically evaluated the role of various scaffolds on the learning of ill-structured problem solving. We propose to draw on this literature while choosing appropriate scaffolds for each step of estimation problem solving. For example, the use of concept mapping in a TELE for the learning of problem solving has been investigated extensively (Stoyanov and Kommers 2006; Hwang et al. 2014; Wu and Wang 2012) and found to be effective for learning. Similarly, the roles of different types of question prompts (Ge and Land 2004) employed as scaffolds at each step of the instructional design for ISP solving has been studied and found to improve learning significantly. There are similar results in the field of engineering education regarding research-based TELEs, strategies and scaffolds for teaching-learning of engineering problem solving (Woods et al. 1997; Wankat and Oreovicz 2015; Bozic et al. 2014; Kalnins et al. 2014; Williams and Ringbauer 2014; Shekar 2014; Zheng et al. 2013; Blowers 2009; Basu et al. 2015; Heidweiller et al. 2011) from which we identified strategies and scaffolds that may be relevant for learning estimation as well.

Thus, the literature on the teaching-learning of engineering and general ISP solving offers us research-based guidelines on what types of scaffolds might support the learning of certain aspects of estimation problem solving, e.g. the use of question prompts for elaboration and reflection. However, the specific activities and the instructional design for learning estimation problem solving is not clear from this literature. On the other hand, within the literature on estimation, there are several heuristics that instructors have used to teach estimation, but they have not been substantiated by empirical evidence. In this work, we bridge this gap by systemically designing and empirically evaluating a TELE for estimation. In the next section, we describe our approach to the design and evaluation of the TELE.

## Design-based research

The broad research goal of this work is to understand how to support learning of estimation problem solving using a TELE. Thus, our goal is twofold: firstly, to design a TELE that leads to the learning of estimation problem solving among learners and secondly, to understand the mechanisms by which learners’ interactions with the TELE leads to this learning. Design-based research (DBR) has “both a pragmatic bent—“engineering” particular forms of learning—and a theoretical orientation—developing domain-specific theories by systematically studying those forms of learning and the means of supporting them” (Cobb et al. 2003). Therefore, we chose DBR as our research methodology.

**Fig. 1:**
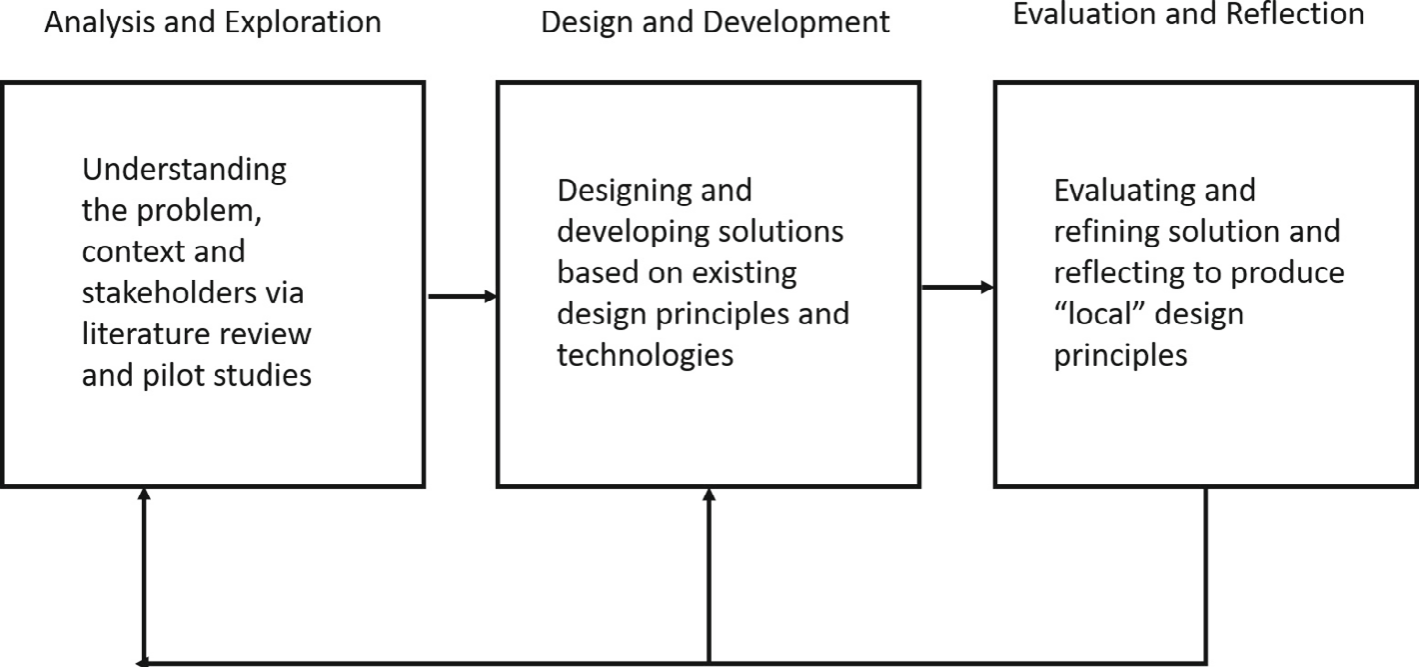
Design-based research as applied in this research work. This figure describes how we applied design-based research methodology in this work

DBR (McKenney and Reeves 2014; Barab and Squire 2004; Collins et al. 2004) (Fig. 1 adapted from McKenney and Reeves (2014)) begins with a detailed analysis of the problem, the context and the participants. This includes an analysis of existing solutions in order to address the problem, perhaps in other contexts and with other participants. It often includes pilot studies and/or ethnographies of the context and participants in order to understand the specifics of the context and the requirements of the participants. Designers and researchers then draw from these theoretical and empirical findings in order to create preliminary LE designs which are then evaluated using various qualitative, quantitative or mixed methods in order to understand the mechanisms by which learning happens in the LE. This is followed by reflection on these learning mechanisms in order to identify how the learning effectiveness of the design could be improved and finally produce design principles or local instructional theories (Cobb et al. 2003). By local instructional theory, we mean a domain-specific instructional theory or a “humble” learning theory that describes how learning happens in our specific context using our designed LE (Cobb et al. 2003). Next, we describe each phase of the first cycle of this DBR project.

## Problem analysis

Broadly, our solution approach is to design a TELE that teaches learners a process of solving estimation problems. We began our research by understanding the process of solving estimation problems.

### Expert study to identify estimation process

In order to understand the detailed process of solving estimation problems of the kind we are interested in, we performed a study of experts (Kothiyal et al. 2016). Our goal for the expert study was to abstract out a systematic process which experts apply in order to solve an estimation problem. We performed a cognitive ethnography (Williams 2006) of two experienced engineers specializing in electrical engineering. These experts are faculty members at our institute and have several years of industry experience as well and active research programmes in their respective areas of research. Each expert solved three estimation problems, and we used multiple data sources to capture their solution process including video recording, screen captures, researcher observations, participant artefacts and stimulated recall interviews. We applied the methods of multimodal data analysis (Hutchins and Nomura 2011) to identify how expert engineers employ cognitive processes and external resources to solve the estimation problem.

**Fig. 2:**
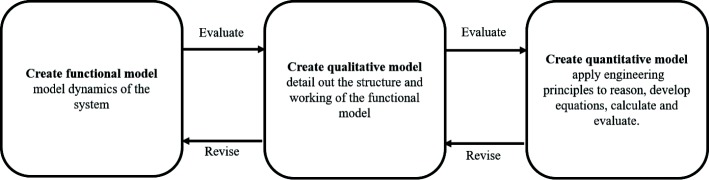
Model-based estimation process. This figure describes the estimation process followed by experts

We identified that experts follow a three-phased progressively higher order modelling process at the end of which experts have a simplified equation that can be used for estimation (Fig. 2). We found that experts begin the estimation process using their preliminary knowledge of how the system functions (functional model of the given problem) and then progressively detail this model until it is sufficiently rich enough for calculating an estimate (qualitative and quantitative models). Further, experts evaluate whether their models at each stage are accurate, complete and yet useful for estimation and plan the rest of the estimation.

The cognitive processes used for modelling include mental simulation, connecting to conceptual knowledge and prior experience, and manipulating external representations such as figures, equations, videos and simulations. They examine whether they can simplify the model by making appropriate assumptions and approximations without compromising on accuracy to a great extent. They also examine whether the critical factors which dominate the system performance in the limiting case are considered or not. Finally, they ensure that the model is in terms of known parameters, making it straightforward to calculate the estimate. While it was known from the literature (“Estimation” section) that estimation is done by creating models, this study showed us the systematic process that experts apply in order to progressively reduce the complexity of the real-world system and create an equation. It showed us how experts make assumptions, where they use mental simulations, conceptual knowledge and external representations. This helped us identify that learners must be scaffolded and provided affordances for doing similar actions.

### Learner study to identify challenges

The exact aspects of estimation problems that learners find difficult and hence need to be provided scaffolds for will depend on their conceptual knowledge and estimation experience. Knowing the difficulties, we can identify from the literature empirically validated scaffolding principles to help learners overcome these difficulties. In order to understand the difficulties learners face while solving the kind of estimation problems we are interested in, we performed a lab study of six first year undergraduate learners (Kothiyal and Murthy 2015) who had the conceptual knowledge necessary to solve the estimation problems we gave them. Each learner individually solved three estimation problems while we provided scaffolds to each learner when they encountered difficulties. The conversations between the researcher and learner were recorded and analysed using thematic analysis (Braun and Clarke 1996) in order to identify the category and degree of difficulty faced by the learner. In addition, we also identified the reasons for these difficulties.

We found that students had difficulty in applying the fundamental concepts of science and engineering, whose knowledge they already had. Specific to estimation, we found that students have difficulty with the open-ended nature of the problem, making assumptions, quantity estimation and assessing numerical values. These were aligned to the difficulties identified in the “Teaching-learning of estimation and other ill-structured problems” section. Further, we explored the difficulties faced by students from an instructors’ perspective. We interviewed an experienced engineering practitioner and instructor and confirmed the difficulties faced by the students while solving the estimation problems and identified additional difficulties, namely, identifying significant/insignificant parameters in the operating conditions and identifying and estimating inefficiencies in the system. Therefore, learners must be scaffolded in these aspects of estimation problems that they find challenging.

### Pedagogical foundation of the TELE

#### Model-based learning

From the expert study, we identified the systematic model-based estimation process that we want students to learn in our TELE. Model-based learning, which involves students creating models of a problem or concept or phenomenon, has been extensively adopted as a teaching-learning strategy in science, engineering and mathematics (Lehrer and Schauble 2000; Wilensky and Reisman 2006; Hamilton et al. 2008; Sun and Looi 2013; De Jong and van Joolingen 2008; Jonassen et al. 2005; White and Frederiksen 1998; Blikstein 2012; Lesh and Lehrer 2003; Basu et al. 2013). The learning goals for which model-based learning have been employed include improving conceptual understanding (Jonassen et al. 2005; Lehrer and Schauble 2000), improving scientific inquiry (Sun and Looi 2013; De Jong and van Joolingen 2008), improving understanding of complex systems (Blikstein 2012; Wilensky and Reisman 2006) and improving students’ computational thinking skills (Basu et al. 2013). Many TELEs have been designed based on model-based learning such as (Slotta and Linn 2009; Wilensky and Reisman 2006; Govaerts et al. 2013; Sun and Looi 2013; Avouris et al. 2003; White et al. 2002; Fretz et al. 2002; Swaak and De Jong 2001) with different pedagogical strategies and affordances in order to scaffold the model construction and learning processes, such as variable manipulation simulations, modelling tools such as causal mapper and equation builder, collaboration via chatting or online discussions, process maps, drawing tools, explanations and structured tasks. Within model-based learning, it has been found that model order progressions (Sun and Looi 2013; Mulder et al. 2011) where students create progressively more sophisticated models are an effective scaffold for students to create quantitative models (equations) and improve their learning. In these interventions, students are provided with appropriate affordances for each stage of modelling (Sun and Looi 2013), such as causal maps and simulations for qualitative modelling (Lindgren and Schwartz 2009; Swaak and De Jong 2001). Thus, we conjecture that for estimation, it will be productive to guide novices through a progressive modelling process ending with the creation of quantitative models, namely, equations for estimation. So, we choose model order progressions as the pedagogical foundation of our estimation TELE, with three phases of modelling, namely, functional, qualitative and quantitative (Sun and Looi 2012; Mulder et al. 2011). The expert study also showed that experts used mental simulation throughout the modelling-based estimation process, but learners have difficulty with this (Hegarty et al. 2003). Hence, we propose to have a simulator to support learners in visualizing and simulating the mechanical system described in the estimation problem.

#### Scaffolding modelling-based estimation

In order for students to do and learn estimation, their interaction with the TELE has to be carefully designed such that the TELE scaffolds their doing and learning process. There are several frameworks which define the types of scaffolds needed for complex, ill-structured tasks in TELEs (Basu et al. 2015; Quintana et al. 2004; Kim and Hanna 2011). The literature suggests that the doing of complex tasks be scaffolded by the complementary mechanisms of structuring and problematizing (Reiser 2004). Ill-structured problem solving literature suggests that in order to obtain a good solution, the solver must periodically evaluate their solution and plan their solution process (Jonassen 1997). Elaboration question prompts have been successfully used in ill-structured problem solving to get students to elaborate and explain their thinking (Ge and Land 2004). Further, at the end of the inquiry process, solvers’ reflection on the entire process has been found to be productive for learning (Quintana et al. 2004). Research shows that students must be scaffolded in order to articulate and reflect on their inquiry (Quintana et al. 2004) and problem solving (Kim and Hanna 2011). In scientific inquiry, expert guidance and opportunities for epistemic reflection (Quintana et al. 2004) help students construct scientific explanations and learn about the epistemic aspects of inquiry (Sandoval and Reiser 2004). Further, research has shown that external representations such as concept maps (Hwang et al. 2014), knowledge maps (Lee et al. 2005), dual maps (Wu and Wang 2012), conceptual organizers, process maps, argument maps and causal maps (Quintana et al. 2004; Slotta and Linn 2009) are effective for learning ill-structured problem solving and scientific inquiry. These representations facilitate process management, model building and sense-making. We propose to use a combination of appropriate scaffolds such as these in our TELE.

## Design of the technology-enhanced learning environment

From the expert study, we identified that experts follow the three-phased progressively higher-order modelling-based estimation problem solving process. These results, along with the literature on the successful use of model order progressions in inquiry learning, were used in the design to structure the estimation process as a progressively higher-order modelling process with tasks for each phase of the process. Next, the identification of the use of mental simulation and external representations by experts extensively during estimation led us to incorporating a simulator and modelling affordances such as a causal map within MEttLE to support these cognitive processes among novices. Finally, in order to mitigate learner difficulties related to the specific aspects of estimation such as understanding the open-ended nature of the problem, identifying significant parameters, making assumptions, quantity estimation and assessing numerical values, we incorporated scaffolds in MEttLE to support novices in these aspects, as recommended in ISP solving literature. These include question prompts for elaboration, evaluation and reflection (Ge and Land 2004) and hints regarding expert practice (Quintana et al. 2004) at appropriate points in the process.

We designed a technology-enhanced learning environment called modelling-based estimation learning environment (MEttLE) based on progressively higher order modelling. We call this learning design as “Progressively higher-order modelling with evaluation and reflection” (ProHOMER). In ProHOMER, the estimation process for each estimation problem is structured (Reiser 2004) into five tasks, namely, functional, qualitative modelling, quantitative modelling, calculation and evaluation. The three modelling tasks each have sub-tasks of create a model, evaluate the model and plan the next steps. The goals of the modelling tasks are:

1. Functional modelling: Learners describe how the system works, i.e. what are the various parts of the system and how these parts are connected together to generate its functioning.
2. Qualitative modelling: Learners identify the parameters affecting power, the parameters (system performance requirements and external parameters) which have a large effect and which can be ignored in the operating conditions, and the qualitative relations between power and those parameters.
3. Quantitative modelling: Learners use conceptual knowledge to create an equation connecting power and the previously identified parameters, incorporating the inefficiencies of the system and making assumptions and approximations in order to simplify the analysis (since the goal is only to get an approximate answer).

**Fig. 3:**
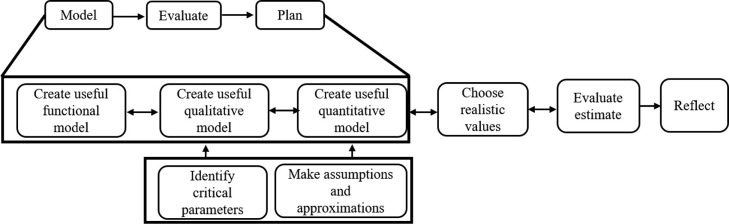
Workflow of MEttLE. This figure describes a possible path that a learner may take while solving a problem in MEttLE

In the calculation task, learners choose realistic values for the equation parameters and calculate an estimate. In the evaluation task, they evaluate the estimate on the basis of two independent criteria, namely, correctness to order-of-magnitude and on comparison with known values. At each modelling stage, learners evaluate their developing models and plan the rest of the estimation process using this model. After obtaining an estimate, learners reflect on their estimation problem solving process. The tasks are problematized (Reiser 2004) using focus questions described below. A possible workflow of a learner working in MEttLE is shown in Fig. 3.

**Fig. 4:**
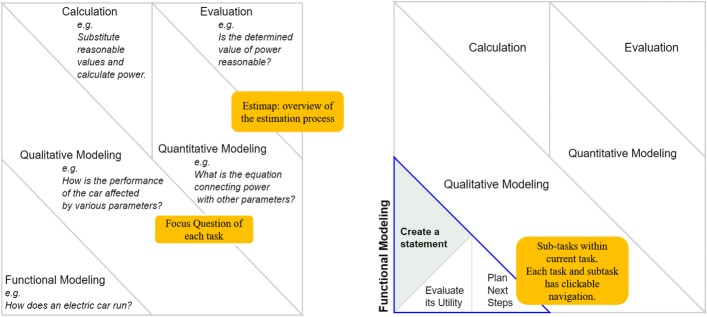
Process management feature “Estimap” of MEttLE. This figure shows the top level and detailed views of the “Estimap” feature of MEttLE

Given the ill-structured nature of the problem, we opted for an open-ended intervention in which the sequence of the five tasks is not prescriptive but suggested using the feature of “Estimap” (Fig. 4) which is the central process management feature of MEttLE. Learners are free to choose any task/sub-task to do at any point in the estimation problem solving process and can do the (sub-)tasks as many times as they wish, but the problem is considered complete only when their estimate passes the specified evaluation criteria. Once a learner selects a task, however, he/she has to complete all the sub-tasks of that task before proceeding to the next task. There are affordances available for learners to achieve the goals of each (sub-)task which are described in detail below. In addition, MEttLE has general purpose tools for information, drawing and taking notes, simulating the system (Fig. 6) and a calculator, which are always available to the student. The features are elaborated below.

**Fig. 5:**
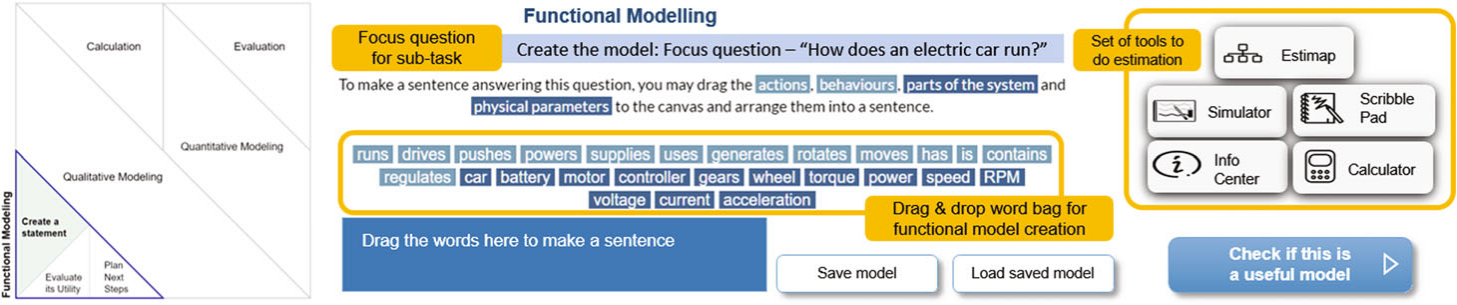
General structure of each task/sub-task. This figure shows the general structure of each task and sub-task in MEttLE

1. Estimap: This is a process management scaffold (Fig. 4) showing the tasks of the ProHOMER learning design. The learner can click on any task to see its sub-tasks and begin doing the task. The Estimap offers the learner the flexibility to choose tasks and thus his/her solution path. There was a short introductory video describing what the Estimap depicted.
2. Modelling tasks: Each modelling task (Fig. 5) is divided into three sub-tasks, namely create, evaluate and plan sub-tasks. The overall task has a focus question which brings attention to the goal of the task. For example, the focus question for functional modelling is, “How does an electric car run?” In addition, the create sub-tasks also have a modelling affordance. For creating a functional model, the affordance is a drag-and-drop word bag containing a set of words describing actions, behaviours, parts of the car and physical parameters. Learners select words from this set to create a sentence answering the focus question, thus creating the functional model. The modelling affordance for qualitative modelling is a causal map creator, and for quantitative modelling, it is a drag-and-drop equation builder. The evaluate sub-task has “model evaluation questions”, such as for evaluating the functional model, there is the question “Does the model describe how power is generated and used in this system? Explain”. Similarly, in the plan sub-task, there are two types of questions, the “estimation practice questions” (e.g. “What performance requirements from the car will dictate the power requirements and choice of motor?”) and planning questions (“What steps will you follow to determine power using this model?”).

**Fig. 6:**
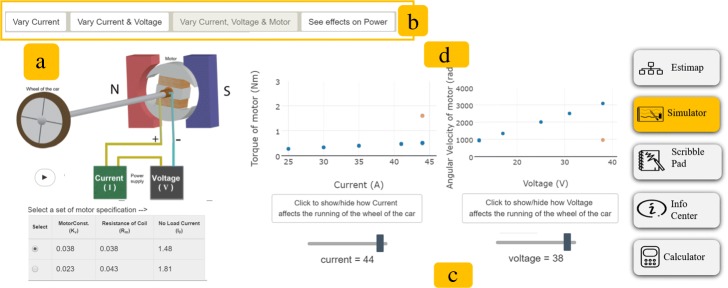
Simulator. This figure shows the various features within the simulator of MEttLE. **a** Simulation of problem system, **b** Progression of exploration activities, **c** Change relevant physical parameters, **d** Graphs of relevant physical parameters
3. Calculation task: In this task, the learner selects appropriate numerical values for the parameters in their equation and calculates the power estimate. Learners are prompted to think about how reasonable the numerical values are and justify them. In order to aid them in this, they are provided scaffolds as “hints” that they can use to assess if their chosen numerical value is reasonable.
4. Evaluation task: In this task, the learner evaluates whether the final estimate is of the right order-of-magnitude and comparable to known values by answering “numerical evaluation questions”, such as “What order of magnitude of power do you expect is needed to run a car? Is the power you determined of the expected order of magnitude? If not, what could be the reason?” The learners use the prompts to self-assess their estimate and are currently not provided any feedback by METTLE.
5. Simulator: This consists of a variable manipulation simulation (Fig. 6) showing the problem system (a in Fig. 6), the parameters affecting power in the system (Fig. 6a) and graphs showing the variation of power with each of these parameters (Fig. 6d). The parameters that can be varied are presented to the student one-by-one in order to constrain their exploration productively (Fig. 6b).
6. Scratch pad: In this space, learners can take notes and make diagrams if they feel the need to, for instance, while they read or use the simulator.
7. Info centre: This space has reference material including documents/webpages/videos for the student to familiarize themselves with the problem system.
8. Reflection activity: In this activity done after the learner has obtained a reasonable estimate, the student answers a set of questions asking them to reflect on their own estimation process, the tasks that they did and the sequence in which they did them. An example question is, “Why did I need to do all these steps (of estimation)?”

Of these features, the ones which directly support learners in doing and learning the modelling-based estimation process are the Estimap, modelling tasks, calculation task, evaluation task, simulator and reflection activity. These are the salient features of MEttLE; while the remaining, namely, scratch pad and info centre, are supporting features, since the former is an affordance for note taking, while the latter provides conceptual knowledge rather than process guidance.

## Evaluation of MEttLE

As is the norm in DBR, our goal for the evaluation was twofold: firstly, to understand how learning happens in MEttLE, which will contribute towards the local learning theory, and secondly, to understand the design limitations, which will contribute towards design refinement. Specifically, to develop a local learning theory, we need to understand what the students learned about estimation and how the learning design and features of MEttLE helped students solve the estimation problem and learn estimation. We had three research questions related to this:

1. How do learners use the features in MEttLE to solve the estimation problem?
2. How did the learning design and features in MEttLE support student learning about estimation?
3. What do students learn about estimation after working in MEttLE?

Secondly, for the redesign, we need to identify what changes/additional features are needed in MEttLE to further improve the learning of estimation. This goal is aligned with the reflection phase of DBR.

### Research design and participants

We performed a lab study, and participants were 11 learners (1 female) from second year undergraduate engineering programmes, 8 from Mechanical Engineering and 1 each from Aerospace Engineering, Chemical Engineering and Engineering Physics. They were selected by convenience and then purposive sampling in order to cover a range of backgrounds—different departments and engineering curricula—in order to increase the likelihood of observing diverse behaviours. Further, we selected participants who had participated in non-curricular technical activities such as engineering design competitions. The reason was that we had earlier found that solving estimation problems requires a fluidity with the application of engineering concepts (Kothiyal and Murthy 2015) which develops with technical experience, and we did not want lack of this fluidity to be a barrier to their estimation in MEttLE. The average age of learners was 20 years, and they were familiar with the use of computers through other courses and labs in their curriculum. One participants’ data was not used as the audio recording was of poor quality and could not be transcribed.

### Materials

Since this is the first iteration of DBR, we want to study rich, minute details of the interaction which will give us insight into learner estimation problem solving, as well as how a pedagogical design in a TELE can support it. So, we incorporated only one problem in MEttLE. The problem was “You are participating in an electric car race in which you are required to design an electric car of weight 7 kg with wheel diameters of 4 in. that can accelerate at 1 m/s^2^ and traverse a track of 10 m without burning out. Estimate the electrical power needed to achieve this performance and the specifications of the motor you will need.”

We decided to choose a power estimation problem as power estimation is important to do before designing any system. While deciding on the problem context, we tried to select one which students would be able to relate to and would be motivated to solve. We conjectured that an unknown problem context in addition to the uncertainty inherent in an estimation problem would overwhelm the students and lead to frustration. As it was found that a lot of students participate in robotics and car racing competitions, we created the above problem for MEttLE. The problem covers all aspects of estimation (mentioned in the “Estimation” section), namely coming up with an order of magnitude number, identifying important parameters, making approximations, quantifying losses and choosing and assessing reasonable numerical values. Thus, this is an appropriate and relevant problem to teach students estimation with.

### Data sources

As the purpose of this study is exploration, we collected multiple sources of qualitative data in order to examine learner performance (Table 1). To answer RQ1, the data sources were (i) individual semi-structured interviews with learners after their interaction with MEttLE (audio-taped and transcribed), (ii) screen recording of learners’ interaction with MEttLE using the screen capture software CamStudio (https://camstudio.org/), (iii) learner-generated artefacts before and during their interaction with MEttLE and (iv) video recording of the student while they worked in MEttLE. As this was the first cycle of DBR and MEttLE had only one problem, to answer RQ3, we did not measure learning via a post-test. Instead, we used student responses during their interview to evaluate what they learned about estimation.

**Table 1:** Data sources used to answer the RQs

Data source	Semi-structured interviews	Screen recording	Learner-generated artefacts	Video recording
RQ				
1	x	x	Triangulation	Triangulation
2	x	x	Triangulation	Triangulation
3	x			Triangulation

### Procedure

The overall procedure for the research study consisted of the following steps:

1. Initial briefing: We briefed participants about the study and its objectives and obtained their consent for recording their audio, video and computer screen.
2. Pre-test: Participants solved an estimation problem on paper, independently and without any researcher guidance. However, they were allowed to use the Internet to search for resources/information/concepts that they needed. They were allowed as much time as they needed to solve the question. The problem given to the learners was “You are participating in a competition in which you are required to design an electric car of weight 5 kg with wheel diameters of 5 in. that can accelerate at 1 m/*s*^2^ and traverse a track of 25m without burning out. Estimate the electrical power needed to achieve these specifications.” which is similar to the problem they would solve in MEttLE.
3. Interaction with MEttLE: Participants interacted with MEttLE and solved the estimation problem mentioned earlier. During this interaction, they were not allowed to use the Internet. However, they were free to use all the resources in MEttLE all the time and ask the researcher any questions regarding how to use the resources MEttLE.
4. Individual semi-structured interview: After the interaction, we interviewed the learners using a stimulated recall protocol wherein their screen capture was played back to them and we asked them to describe what they did at each point in the solving process and reasons for their actions. In addition, we asked them questions about the nature of estimation and the estimation process.

### Data analysis

In order to baseline participants’ estimation performance, we assessed their pre-test solutions and solutions in MEttLE using the following set of criteria which assess both the product and estimation process followed. These criteria were obtained by synthesizing the criteria for good estimation described in literature (Mahajan 2014; Linder 1999; Adolphy et al. 2009; Kothiyal et al. 2016).

1. Estimate is of the right order of magnitude.
2. The important parameters which affect power in the system are identified.
3. Appropriate assumptions and approximations are made.
4. The losses or inefficiencies in the system are considered.
5. Reasonable numerical values are chosen for parameters involved in the estimation.

In order to answer RQs 1 and 2, we applied interaction analysis (Jordan and Henderson 1995) of learners’ interactions with MEttLE. We began by creating summaries of learners’ estimation processes in MEttLE using researcher notes, learner artefacts (generated on paper and in MEttLE) and transcripts of student interviews and viewing the screen captures and video recordings. Next, we used “Elan” (https://tla.mpi.nl/tools/tla-tools/elan) to create annotations of the actions done by learners while solving the estimation problem, both on each screen of MEttLE and off screen on paper, using their screen captures and video recordings. Finally, we added the explanations of their actions given by them during the interview, to the corresponding annotation of the action. Threading the actions and explanations together, we created descriptions of learners’ estimation process as it happened sequentially in time. From these descriptions, we abstracted out the ways in which learners interacted with various features in MEttLE (RQ1) and the roles of the features in MEttLE on their learning of estimation (RQ2).

To answer RQ3, we employed thematic analysis (Braun and Clarke 1996) of student interviews. Thematic analysis is an appropriate method for this research question because our goal is to explore the range of estimation learning experiences existing within our data. The thematic analysis was done by the first author of this paper, a trained researcher in qualitative methods in educational technology. Following the methods of inductive thematic analysis, we first transcribed the interviews and familiarized ourselves with the data. At this stage, in order to get a better understanding of student responses and their context, especially when they referred to their actions or created artefacts in MEttLE, we studied the relevant screen capture, video or artefacts and added these annotations to the transcripts. Then, we generated initial codes across the entire data set and collated related codes into categories and themes. Next, we reviewed the themes against the raw data for consistency and generated an analysis map. Finally, we refined our themes by examining their details and created clear descriptions of them.

## Results

While solving the pre-test problem, before interacting with MEttLE, we found that five out of ten students obtained an estimate off by an order of magnitude. While the remaining obtained an order-of-magnitude estimate, they were not able to satisfy any of the other four criteria for good estimation. All except one student began by thinking directly of equations relating power to the given quantities. The estimation on paper was an exercise in searching for the right equation, in their minds and/or on *Google*, without understanding the power requirements of the car. Only one of the students began by considering the working of the electric car and thought of the inefficiencies in the system. The rest only considered the mechanical aspects of the car and created an equation. Further, they were unable to identify which parameters would dominate, which could be ignored and which approximations were appropriate. While solving in MEttLE, students improved their performance and obtained an estimate of the right order of magnitude by considering the correct parameters affecting power, applying conceptual knowledge appropriately to create an equation and choosing reasonable values for the parameters in the equation.

There were modelling tasks/sub-tasks and affordances for students to create and revise models, along with the calculation task and tools of simulator, scratch pad, information and calculator for students to solve the estimation problem. To answer RQ1, we focus on how learners used the Estimap, modelling tasks and affordances, calculation task, evaluation task and the simulator, as these are the salient features for solving, while others are supporting features. Similarly, to answer RQ2, we investigate the effect of the task/sub-task structure along with their focus questions, the Estimap and the metacognitive scaffolds, as these are the salient features for learning about estimation.

### Answering RQ1: “How do learners use the features in MEttLE to solve the estimation problem?”

#### Modelling questions and affordances

All learners used the modelling questions (focus questions, model evaluation questions and estimation practice questions) and affordances to create the functional, qualitative and quantitative models and MEttLE supported a diverse set of productive actions for each phase of modelling. In functional modelling, learners used the words (components, parameters, actions and behaviours), the information resources and the simulator given in MEttLE in order to create the model, which they refined based on the model evaluation questions. Four learners used their prior experience of cars, their imagination of how a car works along with the set of words given to create a complete and useful model. Six learners read the material given, watched the video and explored the simulator in order to effectively use the words to create a complete and useful model. In qualitative modelling, learners created a causal map describing the relationships between power and the parameters which might affect it via multiple interaction paths. Two learners used their conceptual knowledge to create a causal map, while eight learners explored the tabs in the simulator, changed the variables and observed the graphs in order to create the causal map. Learners often iterated between modelling and evaluation in order to create a comprehensive causal map capturing the relationships between power and all the electrical and mechanical parameters.

In quantitative modelling, learners were given a set of parameters and mathematical relationships in MEttLE. In addition, their causal map, the simulator and information resources were available to them. One learner strategy used by four learners was to look at their causal map and apply conceptual knowledge to create an equation. Another strategy (three learners) was to use conceptual knowledge and the given parameters to create the equation and the simulator to verify the validity of the equations. This was described by S10, “This is one of the best parts, I can say because I had all the parameters that I needed, this helps me out that I have to build an equation from this parameters because the others aren’t that necessary, these are the most important parameters, and in a way I thought of voltage and current because only given in the parameters, otherwise only going the direction of mechanical power, but then with these parameters, I realized that I need to link it with the voltage and current required.” The third strategy (three learners) was to create an “empirical” equation for power using the graphs in the simulator and verifying it using the given parameters as described by S5, “Yeah, but I actually didn’t know about the formulae relating I, tau and omega. So, in this case, I do have an idea, of the case you mentioned, I do have an idea about the formulae, but in that case, I didn’t have an idea, so I had to think, create an idea from the data that was given in the simulator.” Regardless of the strategy, learners were able to create an equation for power in terms of mechanical and electrical parameters.

Four learners did qualitative and quantitative modelling together, creating the equations as they identified the qualitative relationships and felt that the two cannot be separated as S7 said, “When you make some changes, like to apply readily, you have to shift; you can’t just do everything qualitative and then after that do the quantitative formula. So I was doing it simultaneously like if you found something, apply it somewhere. And actually, I wrote it in the scribbled notes also, the relations that I found, I tried to make the formula and also I wrote it in the notes section.” Thus, we see that MEttLE afforded different interaction paths to learners to create useful and complete functional, qualitative and quantitative models. Depending on their conceptual knowledge and via their preferred interaction paths, learners were able to create the progressively higher order models and solve the estimation problem.

#### Simulator

The primary purpose of the simulator was to facilitate learner visualization and simulation of the behaviour of the problem system and identifying the qualitative relationships between various parameters, since we conjectured that learners will have difficulty doing this via mental simulation. Learners recognized these benefits of the simulator; the simulator provided a visualization of the effect of different parameters on the behaviour of different parts of the system as mentioned by S10, “Like for such cases, I feel that we need to have some visualization about it, like in this we got a simulator, by which I was able to visualize how it is changing the different parameters, so, same way for solving a problem, we need to have a visualization or some factor that helps you that my this parameter is being affected by so and so parameters, so, it helps you to establish some relationships between the two.” Further, learners also reported that initially, they had only thought of the mechanical power and the factors that affected it, but after interacting with the simulator, they realized how the mechanical parameters are related to the electrical parameters. Articulating this, S5 reported, “That would have been a little difficult to identify as in you are saying that current correlation with torque and that would have been a little difficult to identify because we don’t have any prior experience, working with motors, that much.”

While creating the causal map, some learners followed a systematic approach, varying one variable at a time, observing the changes and making notes; others were less systematic in their exploration and explored only a few variables and tabs before creating the map and then revising the causal map based on the model evaluation questions. We observed multiple additional uses of the simulator by the learners. Firstly, learners used the simulator to create equations after identifying the qualitative relationships. Secondly, learners used the simulator in the quantitative modelling stage to “curve fit” an equation for power using the various graphs shown. In this manner, the simulator served as a bridge enabling students with low conceptual knowledge to do estimation. The third use by learners was to directly read off from the graphs in the simulator the motor parameter values (e.g. current) that would give them the required mechanical performance parameter value (e.g. acceleration). This is standard design practice, and expert engineers often use data sheets of components for the same purpose. Finally, learners also used the simulator to evaluate their final estimate. This was a productive use of the simulator as evaluation is a good practice. Thus, the simulator served as space for integration across the tasks and to solve the larger estimation problem.

### Answering RQ2: “How did the learning design and features in MEttLE lead to student learning about estimation?”

#### Role of structuring the estimation process

In MEttLE, we structured the complex estimation process into a set of five tasks, each with a specific focus question. Learners watched the introductory video and then chose their tasks using the Estimap. They reported that the task structure depicted in the Estimap (Fig. 4), along with the focus questions of each task, made the choice of tasks easier. As described by S1, “So, I went through this [pointing to Estimap] so I knew that evaluation needs to be the last and so...functional modelling was something which I found to be the best part to start with because you need to know how a car runs. Before solving a problem I should know that. After that the qualitative and then the quantitative and the calculation and evaluation.” Although MEttLE was not prescriptive in the task sequence, when faced with all the options of possible tasks, learners recognized the importance of beginning with a model of the working of the car. As a result, all learners chose to begin with functional modelling because it made sense to them that understanding the working principle first would make identifying the parameters that affect power easier. As S7 reported, “I didn’t do it before, but you should know the concept what you are actually doing, you should know that before you actually solve the problem, and you should first analyse it qualitatively, like the relationships and all, that’s actually one of the most important things to do and if we just look at it as a problem and just go through the quantitative part, that way I don’t think it’ll be as beneficial as it was today.” Thus, learners changed their approach from “equation first” while solving the pre-test problem to “functional modelling first” in MEttLE.

Next, we investigated what in MEttLE could have led to this change in learners’ approach. We found that all learners broadly followed the path of functional modelling, qualitative modelling, quantitative modelling, calculation and evaluation. Even though some learners made errors during modelling and had to iterate between the tasks and sub-tasks until they obtained a reasonable estimate, they recognized the utility of this task sequence. When asked why he chose this sequence in MEttLE, S5 reported “The sequence, I don’t know why but that sequence seemed important to me, following the functional modelling and then it felt like it was a natural flow of thinking, that is, first you identify what is the model, then you identify what are the parameters involved in that, then you try to formulate your required output along with the parameters, then you calculate and then compare and the evaluated and iterate it till you get a good estimate.” All learners reported that they were unaware of this sequence before working in MEttLE; however, they would implicitly solve problems in this sequence, although they would often skip steps while doing so. All learners reported that MEttLE made the process explicit for them, and so they shifted to the progressively higher-order modelling estimation process. We argue that the structure of the Estimap, with five task options only, all of which needed to be done in some order, along with their focus questions, provided the complementary mechanisms of structuring and problematizing, which helped students recognize the sequence that would be useful in solving the problem and made the progressively higher-order modelling-based estimation process intuitive and easy to follow.

#### Role of metacognitive question prompts

Learners’ interview responses show that they recognized that they should evaluate their models and numerical values and why they should do so; however, their answers to the evaluation questions were incomplete and inaccurate. The model evaluation questions asked the students to assess whether their models were useful and complete for estimation and revise if not. Further, the estimation practice questions aimed at getting students to assess whether their models were, in addition to being complete, simple enough to get a good estimate. Learners did this assessment and iterated their models based on the model evaluation and estimation practice questions. This helped the learners recognize that periodic evaluation is necessary for estimation, and estimation requires many practical considerations. Further, they learned which questions they must ask themselves while evaluating. However, their responses to several of the model evaluation and estimation practice questions show that they do not know *how to judge* whether their models are useful, complete and yet simple. This is because these judgements require a firm grasp of conceptual knowledge and extensive experience with similar problem systems which students lack. Learners’ responses indicate that their consideration was limited to parameters given in the problem, simulator and/or obvious parameters (e.g. friction), while failing to consider non-obvious, but possibly important parameters (e.g. drag). We found that learners had similar difficulties in assessing their numerical values. Again, most learners were unable to make such judgements, and their responses showed that they had very poor intuition for the numerical values and were unable to reason about them.

The planning questions (in the plan sub-task) were intended as an integration activity to get learners to connect what they had done to what they will do next and learn how to monitor and plan their estimation process. However, we found that they did not serve this designed purpose. Only S5 reported the purpose of planning sub-task of each task as, “...the questions they told me to think of certain things in what I had done, so suppose it was functional modelling, then what I had actually done and what I can do in the next phase, so, what was lacking in what I had done, is addressed in those questions, what I am doing next helped me to link the two, like functional modelling to qualitative modelling, like I could relate the two because of the planning phase.” Others perceived them as an assessment or a hindrance to their estimation flow as reported by S1, “...I would have thought that plan next steps is not necessary like I’m going in a particular way if I just follow it I’ll just reach that place.”

The reflection activity was intended for learners to reflect on the steps in the estimation process and understand the process better. It helped reiterate to learners the importance of first understanding the system working and the stepwise process, as S6 wrote in response to the reflection question, “Firstly I looked up at the theory related to the topic. Then, created a flow chart that gave a relation between all the parameters. Then created an equation that would satisfy the relations. Finally used the equation to find out the power for the given values of parameters.” However, some learners (four out of ten) either did not respond at all or responded superficially to the reflection questions, which showed that they did not think deeply about the role of each task and sub-task in the estimation process. Still, when asked about the value of reflection during the interview, three out of these four learners reported that it was a valuable exercise as exemplified by S8 here, “Yeah, the effect [of the reflection] was that I would actually know that whether I am using these steps while solving any problem in the future, I feel that these are the things that we generally do, but if we don’t know these things step by step, you might end up skipping a step or maybe doing something that is not required.” Thus, we argue that along with structuring, the reflection activity consolidated the learning of the process of estimation.

### Answering RQ3: “What do students learn about estimation after working in MEttLE?”

#### Theme 1: The three-phased modelling-based process is a systematic way to solve estimation problems

**Fig. 7:**
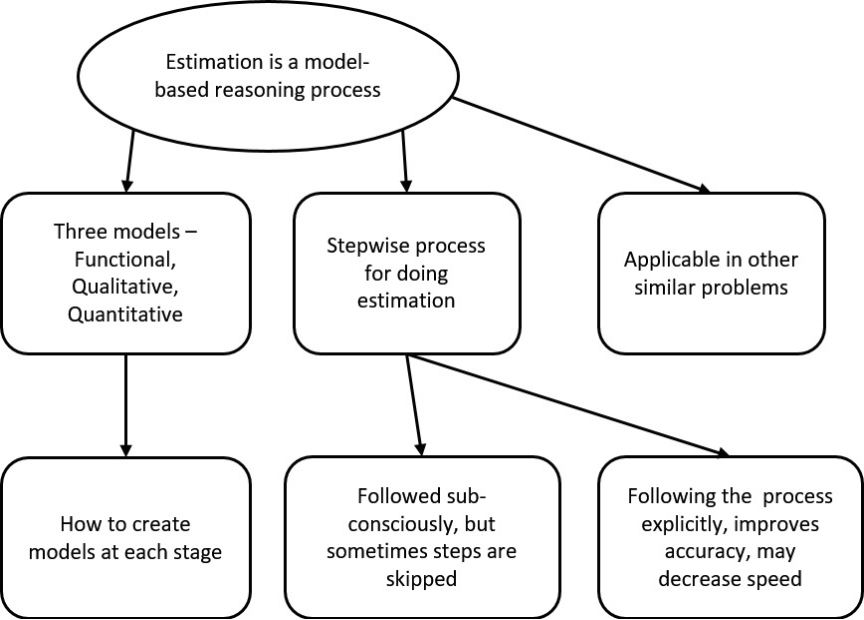
Analysis map of Theme 1. This figure shows the themes identified within Theme 1

The map of this theme is shown in Fig. 7. Learners recognized that the three-phased modelling-based estimation process is a systematic way to solve estimation problems. They perceived that they got a better estimate in MEttLE because they applied this process to solve the estimation problem as reported here by S6, “we’ve never solved any problem in such a systematic manner, like we first study all the theory, then jot down what are the parameters related to it, then try to develop an equation. What we have been accustomed to is like, we would have learned the theory at some point, we already know the formula related to it, we don’t think of first seeing the proportionalities and why this is happening. Here we saw the simulations, we could see the on time relations that would be varying, like when we varied the input current or voltage the torque and acceleration were varying, so, we could see, like we could get an overall view.” Thus, learners perceived that each step of the process contributed towards solving the given estimation problem.

Learners also understood how to create the models at each phase as described here by S4, “You first build up a functional model, that you think of, you only imagine right, imagine the moving parts, like what goes where, what happens when, what pushes what and all sorts of things. You kind of think of an imaginary model, you try to think of an animation, and then you try to get to the various relationships between the quantities, the, qualitative modelling shows exactly that, then the quantitative modelling, there you actually start writing down the equations and tinkering around with them.” Further, learners realized that they typically follow this process only sub-consciously and so tend to skip steps and that following the process explicitly would improve accuracy in the case of unfamiliar problems as mentioned by S3, “...but if there is something, which I think I don’t know much about that, then, now, I think I should prefer this way, where I would make a model and everything.” Finally, learners reported that the three-phased modelling-based estimation process is applicable to other problems and that they would apply it for similar estimation problems as mentioned by S9, “After engineering, when we go for a job somewhere, we will be faced with such a situation where we will have to design something, so I’ll need an estimate, I’ll need to meet some criteria, so now I know a procedure, a series of steps, which I can apply to get an estimated value, that’s what I learned today.”

#### Theme 2: Evaluation is necessary for estimation

**Fig. 8:**
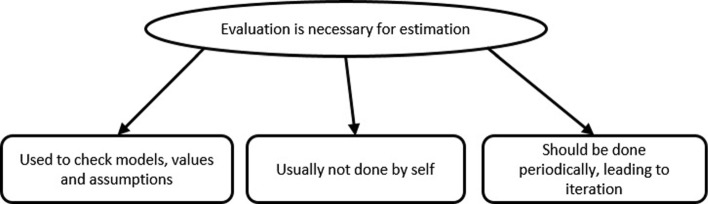
Analysis map of Theme 2. This figure shows the themes identified within Theme 2

The map of this theme is shown in Fig. 8. After working in MEttLE, learners recognized the usefulness of evaluation as described here by S5, “I think just that the evaluation part was very critical, because, if that is not there, we might not be able to identify where we have gone wrong at all, and that helps you go through the cycles faster.” Learners reported multiple benefits to evaluating like focussing on things that may have been missed, iteratively correcting errors and making things explicit. Further, they also observed that when estimating on their own, they usually do not evaluate. Additionally, learners reported that the specific questions in the evaluation sub-tasks and final evaluation task in MEttLE were critical in getting them to check and make revisions to their models and estimates. This is evident from this quote from S3 about checking numerical values “...in the first iteration I found it, this one as 10 Kilo watts, and then I realized my mistake, in that torque thing, because, when I came back to this, I, switched, like just checked what is this, and they said that, you should, you can compare it with the vacuum cleaner.”

#### Theme 3: Estimation requires many practical considerations

**Fig. 9:**
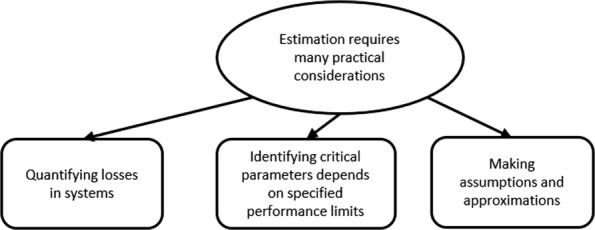
Analysis map of Theme 3. This figure shows the themes identified within Theme 3

The map of Theme 3 is shown in Fig. 9. Firstly, learners recognized that doing estimation requires thinking of several practical aspects of engineering problems. One of these is quantifying losses, which they have difficulty understanding, as S8 describes: “I didn’t even know that there were two different things, we are considering the losses, I didn’t know that...that should be made clear that there are losses considered and by what factor is the difference between the input and output power.”

Secondly, learners recognized that in order to decide which system parameters are important for power estimation, they need to understand the limits of system performance when it is actually working, as S2 says, “So, it depends on what is critical, in the system where I want to put it, so, if I were to say, the current in the system at certain time, should not exceed the maximum current value, then I cannot go with average value, I need to know that at all times the current is below the Imax, it should not be that current is below the Imax, so, it becomes, a system constraint, that in the system which I want to put it, I cannot have something, like an average is below the constraint, but instantaneous can cross it, so, I think my system will decide how I would use it.”

Finally, learners understood the need to make assumptions and approximations because estimation does not require precision, but speed, and that the assumptions and approximations should be reasonable so that they do not cause large errors in the estimate. Learners, however, are unable to make these judgements and may end up making inappropriate assumptions for the wrong reasons, as evident from S5’s reasoning here, “Umm, because I think that the order of those terms is complex. Like when we were trying to study for them, I think the coefficient of drag that you have to calculate, that depends on a lot of things, and to calculate that I need lots of data, so, I left them out.”

## Reflection and discussion

At the end of the first cycle of DBR to design MEttLE, we reflect on the results of our evaluation in order to identify what redesign of MEttLE is needed in order to improve learner performance. The goals of this evaluation were to explore how learners solved the estimation problem in MEttLE and what and how they learned while solving. We found that learners were able to use the resources provided in MEttLE to solve the problem. Specifically, learners used the modelling questions and affordances and the simulator in order to create and revise models while solving the estimation problem. MEttLE afforded a diverse set of productive interaction paths for each phase of modelling and the overall estimation process; it supported expert-like actions, such as using imagination (Kothiyal et al. 2016) to build a functional model and systematic use of the simulator to build a qualitative model, while also supporting novice learners who were unable to use imagination, by allowing them to use conceptual knowledge and information resources in MEttLE in order to build models.

We found that the simulator served as a good tool for visualization and qualitative understanding of the system (Lindgren and Schwartz 2009; Swaak and De Jong 2001) which are necessary for the estimation process. In addition, the simulator was used in several unintended ways, and we found that the simulator served as an integrator across tasks. This role is similar to experts who use mental simulation throughout their estimation process to create, evaluate and refine their models and estimates (Kothiyal et al. 2016). An issue that we observed with the simulator was that its availability drew some learners away from the main task and they spent a considerable time exploring it, trying to understand the relationships between all the parameters rather than doing the modelling tasks. Therefore, we need to further investigate whether the simulator serves as “crutch” or a scaffold that can be faded. We are also considering providing a simplified version of the simulator rather than the current variable manipulation simulator for some parts of the pedagogy.

We found that learners understood that the three-phased modelling-based process is a systematic and useful process to solve estimation problems. This was because of the five-task structure we had provided (Reiser 2004), which was based on model order progressions (Swaak et al. 1998; Sun and Looi 2013) and the focus questions, which problematized the tasks (Reiser 2004) by directing learners’ attention on important aspects of the modelling-based estimation process. Learners described the role of the Estimap in making the MBR process explicit, and this is consistent with the benefits of external representations for process management documented in scientific inquiry (Quintana et al. 2004) and problem solving (Hwang et al. 2014). We argue that the design of Estimap, with five task options and their focus questions, was a *productive constraint* that helped students discover a sequence that would be useful in solving the problem. It was a constraint because learners had to choose one of the five tasks; it was productive because it highlighted the goals of each task and enabled learners to solve the problem. While it could be argued that MEttLE prescribes a structured process for an ill-structured problem, we argue that it is not prescriptive; learners may choose the path they wish to take depending on the problem. Indeed, as some learners mentioned, they would follow the three-phased process beginning with functional modelling for problems which had an unknown system, while they would prefer to go directly to equation building in the case of familiar systems. This is also consistent with expert behaviour (Kothiyal et al. 2016), wherein the first two phases of modelling are done implicitly in a familiar problem. Further, students reported their intention to apply the three-phased modelling-based process to other estimation problems; while this is important, we have not measured student performance independently using a post-test, and hence, these findings need to be validated in future studies.

Learners reported that the model evaluation, numerical evaluation and estimation practice questions provided periodically in MEttLE helped them learn two things: firstly, the importance of evaluating their models and values, and secondly, the practical aspects of estimation. The nature of the questions guided them regarding what to evaluate and what practical aspects to consider. This result is in agreement with research into the role of question prompts (Ge and Land 2004) in ISP solving. However, learners’ responses to the questions were incomplete and inaccurate, indicating that they were unable to make the judgements required in the questions. This ability develops with experience and practice in solving problems with similar systems and comparisons with similar values (Linder and Flowers 2001; Mahajan 2014). Specifically, students need to understand how to apply conceptual knowledge in real-world conditions to make decisions. The current version of MEttLE does not have any guidance on how to think about these aspects of estimation nor do students have any exposure to these in their engineering curriculum. So, we propose to introduce guidance of expert practice (Quintana et al. 2004; Sandoval and Reiser 2004) at appropriate points in the pedagogy. Also, as recommended in the literature, we propose to add small activities and additional resources in order to scaffold learners in choosing and evaluating numerical values (Linder 1999; Mahajan 2014; Shakerin 2006; Dunn-rankin 2001).

Finally, we had intended the planning questions to be the integrator across tasks, helping students keep track of their progress and plan the next steps. However, most learners did not find them useful for this purpose, perceiving them to be assessment and choosing instead to hold their plan in memory, take notes in the scratch pad or use the simulator as the integrator. Hence, we need to re-examine the need for these questions, perhaps incorporating alternate scaffolds for planning such as a checklist of possible tasks, a progress bar or a planning map.

The sample size of this study is small, which is a limitation. However, the larger goal of this evaluation is a rich and in-depth characterization of how students do and learn estimation in MEttLE. The goal was to identify how this learning mechanism can be made more productive by redesign. The current results reveal the nature of learning and learning mechanisms that happened in MEttLE. In the next cycle of DBR, we will modify the design of MEttLE based on these results in order to improve learners’ estimation process and performance. In this iteration, we only asked students during the interview to explain what they understood of the three-phased modelling-based process and describe how they would solve a similar problem that we gave them. From the results, we found that students understood the process. In the next iteration, we propose to fade the scaffolds in MEttLE and test whether they would actually be able to solve another estimation problem by applying the three-phased process.

## Conclusions

Estimation is an important class of ill-structured engineering problems that engineers need to solve at the workplace. However, it has been found that even final year undergraduate engineering students cannot estimate quantities such as force and power. This is because undergraduate students do not have an opportunity to solve such problems because current engineering curricula do not include the learning of estimation. In this paper, we reported on the first cycle of our DBR project to design and evaluate MEttLE, a TELE for estimation. MEttLE has at its core a model order progression-based structured estimation process. The results of our evaluation of this first design showed how learners were able to use the features in MEttlE to solve the estimation problem. Further, students learned the estimation problem solving process, and we identified how each feature in MEttLE contributed to this learning. Reflecting on the results of our preliminary evaluation, we identified several gaps in students’ learning of estimation and possible reasons for these gaps. These will be addressed via redesign in the second cycle of DBR and evaluation of how learning improves. We will also identify the learning mechanisms which enable students to use MEttLE to learn estimation problem solving.

The results of the evaluation of this first cycle offer some evidence-based guidelines to teachers who want to teach estimation in the classroom. Students learn estimation by learning to apply the three-phased MBR process which begins with functional modelling by mental simulation. So, teachers can scaffold students to apply this structured process while estimating. Further, mental simulation is a cognitive tool which enables solvers to visualize the working of the entire system and how different parameters “flow” inside the system. It is required during the first two modelling phases of estimation. Hence, teachers can scaffold learners’ mental simulation by providing appropriate question prompts which guide learners to visualize the working of the system, what is the mechanism that drives it, what are the dominating parameters and so on.Further, teachers should intermittently prompt students to evaluate their models and numerical values. In order to develop students’ sense of numerical values, teachers must make them do several small activities of comparing values of commonly used physical parameters such as power and force. Finally, doing several such engineering problems may improve students’ ability to reason about the practical aspects of estimation problems. We conjecture that incorporating a few such problems in every course can help students attain proficiency in estimation over the course of the engineering programme and become better estimators. It would be interesting to investigate how many and what types of estimation problems students must practice on to gain expertise in estimation.

## Abbreviations

DBR: Design-based research
ISP: Ill-structured problem
MEttLE: Modelling-based estimation learning environment
ProHOMER: Progressively higher-order modelling with evaluation and reflection
TELE: Technology-enhanced learning environment
